# The Functional Network Processing Acute Electrical Itch Stimuli in Humans

**DOI:** 10.3389/fphys.2019.00555

**Published:** 2019-05-15

**Authors:** Hideki Mochizuki, Loren E. Hernandez, Gil Yosipovitch, Norihiro Sadato, Ryusuke Kakigi

**Affiliations:** ^1^Dr. Phillip Frost Department of Dermatology and Cutaneous Surgery, Miami Itch Center, Miller School of Medicine, University of Miami, Miami, FL, United States; ^2^Department of Integrative Physiology, National Institute for Physiological Sciences, Okazaki, Japan; ^3^Department of System Neuroscience, National Institute for Physiological Sciences, Okazaki, Japan

**Keywords:** itch, posterior insula, basal ganglia, functional connectivity, fMRI

## Abstract

The posterior insula (pIns) is a major brain region that receives itch-related signals from the periphery and transfers these signals to broad areas in the brain. Previous brain imaging studies have successfully identified brain regions that respond to itch stimuli. However, it is still unknown which brain regions receive and process itch-related signals from the pIns. Addressing this question is important in identifying key functional networks that process itch. Thus, the present study investigated brain regions with significantly increased functional connectivity with the pIns during itch stimuli with 25 healthy subjects by using functional MRI. Electrical itch stimuli was applied to the left wrist. Similar to previous brain imaging studies, many cortical and subcortical areas were activated by itch stimuli. However, not all of these regions showed significant increments of functional connectivity with the pIns during itch stimuli. While the subjects perceived the itch sensation, functional connectivity was significantly increased between the right pIns and the supplementary motor area (SMA), pre-SMA, anterior midcingulate cortex (aMCC), anterior insula (aIns), secondary somatosensory cortex (SII), and basal ganglia (BG), suggesting that this is a key network in processing itch. In particular, intensity of functional connectivity between the pIns and BG was negatively correlated with itch rating. The functional pIns-BG pathway may play an important role in regulation of subjective itch sensation. This study first identified a key brain network to process itch.

## Introduction

Itch is an unpleasant somatic sensation provoking the desire to scratch. The periphery nerve fibers that transmit this sensation are unmyelinated fibers (C-fibers) and some thin myelinated fibers (Aδ-fibers) (LaMotte et al., 2014). A majority of somatosensory signals conveyed by these peripheral fibers reaches the posterior insula (pIns) via the spinothalamic tracts (STT) and thalamus (Andrew and Craig, 2001; Simone et al., 2004; Davidson et al., 2007; Dum et al., 2009; Craig, 2010; Segerdahl et al., 2015). The pIns further transmits these signals to broad areas in the brain, which eventually generate somatic sensations include the pain, itch and thermal sensations (Craig, 2010; Garcia-Larrea et al., 2010; Isnard et al., 2011; Peltz et al., 2011). Therefore, applying electrical stimuli to the pIns can provoke various somatic sensations (Ostrowsky et al., 2002; Stephani et al., 2011; Mazzola et al., 2012). This evidence demonstrates that the pIns is an important hub brain region that constitutes a brain network in the processing of somatosensory neural signals and this network plays an important role in perceptions of somatic sensations. A previous functional connectivity MRI study found that the pIns increases functional connectivity with various brain regions involved in pain processing during pain and thermal stimuli, indicating that these brain regions constitute a key network to process pain- and thermal-related neural signals from the pIns (Peltz et al., 2011). In itch studies, previous brain imaging studies have identified brain regions activated by itch stimuli including the prefrontal cortex (PFC), supplementary motor area (SMA), premotor cortex (PM), somatosensory cortex, lateral and medial parietal cortex, midcingulate cortex (MCC), claustrum, anrterior insula (aIns), pIns, basal ganglia (BG), the thalamus, and cerebellum (Hsieh et al., 1994; Darsow et al., 2000; Drzezga et al., 2001; Mochizuki et al., 2003, 2007, 2008, 2014; Walter et al., 2005; Leknes et al., 2007; Herde et al., 2007; Valet et al., 2008; Schneider et al., 2008; Ishiuji et al., 2009; Bergeret et al., 2011; Papoiu et al., 2012, 2014; Kleyn et al., 2012; Napadow et al., 2014). However, it is still unknown which brain regions constitute a functional network with pIns in the process of itch. Thus, the aim of the present study was to identify brain regions increasing functional connectivity with the pIns during itch stimuli using functional connectivity MRI.

## Materials and Methods

### Subjects

Twenty five healthy subjects (age: 28 ± 9 years old), 7 of whom were women, participated in this study. Written informed consent was obtained from all subjects. The study complied with the Declaration of Helsinki, and the Ethics Committee of the National Institute for Physiological Sciences (Japan) approved the experimental procedures.

### Electrical Itch Stimulus

An electrical stimulation method was used to evoke the itch sensation in the present study (Ikoma et al., 2005; Mochizuki et al., 2008, 2009). Similar to our previous studies (Mochizuki et al., 2009, 2014), electrical stimuli were applied to the left wrist through electrodes (Vitrode, F-150M, Nihon Kohden, Tokyo, Japan). There were six blocks in a session. Each block was 22.5 s. The interval between the end of a block and the beginning of a following block varied between 40 and 50 s. Continuous application of electrical itch stimuli during a whole block (i.e., 22.5 s) can induce habituation effects such as decay or disappearance of the itch sensation during the stimuli. To avoid this risk, a short duration of itch stimuli (2.5 s) was applied 5 times with interval of 5 s (i.e., 5 s from the beginning of a 2.5 s-itch stimulus until the beginning of a following itch stimulus) in each block. The conditions of electrical itch stimulus were 0.35 mA, (current intensity), 50 Hz (frequency), a 10 m s-pulse width and 125 repetitions of the pulses. To minimize effects related to evaluation of itch intensity on brain activity during each block, subjects were asked to rate average itch sensation of itch stimuli during the six blocks using numerical rate scaling (NRS) ranging from 0 (no itch) to 10 (the worst itch) at the end of the session.

### MRI Measurements and Preprocessing

The MRI experiment was conducted with a 3-T MRI scanner (Allegra, Siemens, Erlangen, Germany) at the National Institute for Physiological Sciences in Japan. For functional imaging during each session, a series of 156 volumes was acquired with T2*-weighted, gradient-echo, echo-planar imaging (EPI) sequences. Each volume consisted of 39 transaxial slices, each having a thickness of 3.0 mm, with a 0.5-mm gap between slices to cover the entire cerebrum and cerebellum [repetition time (TR) × 2,500 ms; echo time (TE) × 30 ms; flip angle (FA) × 80°; field of view (FOV) × 192 mm; 64 × 64 matrix]. Oblique scanning was used to exclude the eyeballs from images. The first three EPI volumes of each session were eliminated to allow for stabilization of the magnetization. Thus, the fourth scan was the first volume. The functional MRI data were analyzed with statistical parametric mapping eight software (SPM8, The Wellcome Trust Centre for Neuroimaging, London, United Kingdom^1^). All EPI volumes were realigned, and the first volume was normalized to the Montreal Neurological Institute (MNI) EPI image template using an affine transformation and a non-linear basis function. The same parameters were applied to all EPI volumes, which were spatially smoothed in three dimensions with a Gaussian kernel with 6-mm full width at half-maximum.

### Itch-Related Brain Regions

Individual activity associated with electrical itch stimuli was identified using the regressor (Duration: 9 scans, Repetition: 6) convolved with a canonical hemodynamic response function (First-level analysis). To make inferences at a population level, individual data were summarized and incorporated into a random-effects model (second-level analysis) (Holmes and Friston, 1998). The statistical threshold for significant change in activity was uncorrected *P* < 0.001 for intensity and family wise error rate (FWE) corrected *P* < 0.05 for cluster (whole brain) for the analysis mentioned above. We also conducted correlation analysis to identify brain regions in that activity is significantly correlated with itch NRS rating (Threshold: uncorrected *p* < 0.001 and *p* < 0.05 FWE corrected for the whole brain).

### Psychophysiological Interaction Analysis of Activity in the pIns

We used psychophysiological interaction (PPI) analyses implemented in SPM (Friston et al., 1997) to search for brain regions showing greater functional connectivity with the seed region (i.e., the right pIns) during itch stimuli. The time series of the blood oxygenation level–dependent (BOLD) signal within a 3-mm radius sphere around the coordinates of the pIns identified in the above analysis (i.e., Itch-related brain regions). The PPI was calculated using the time series data. The interaction term was subsequently reconvolved with the hemodynamic response function. The reconvolved interaction term was then entered as a regressor in a first-level model together with the time series of the seed region and the psychological vector of interest (i.e., regressor used to identify brain region activated by itch stimuli). Then, the models were estimated (first-level analysis). Subsequently, a second-level random effects analysis was performed. We identified brain regions that showed significantly increased or decreased connectivity with the seed region during itch stimuli (threshold: uncorrected *p* < 0.001 with cluster level FWE whole brain corrected *P* < 0.05). As our interest is functional connectivity between brain regions that are significantly activated during itch stimuli and the pIns, the second-level analysis was masked with brain regions significantly activated by itch stimuli. In addition, we investigated in which brain regions intensity of functional connectivity with the pIns showed significant correlations with itch NRS rating (threshold: uncorrected *p* < 0.001 and FWE corrected *p* < 0.05 for the whole brain).

## Results

### Itch NRS Rating and Brain Regions Significantly Activated During Itch Stimuli

**FIGURE 1:**
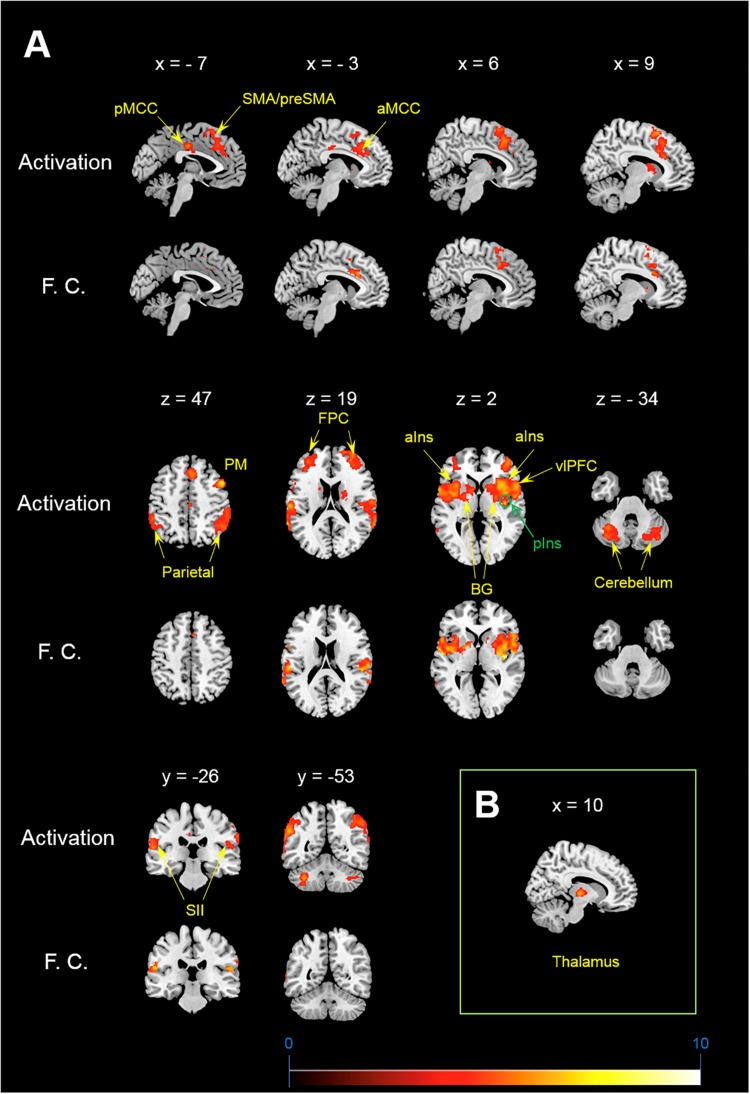
Activation and functional connectivity maps during itch stimuli. **(A)** brain regions showing significant activation (Activation) and significant increment of functional connectivity (F.C.) during itch stimuli. **(B)** Functional connectivity between the pIns and thalamus. MNI coordinate (x, y, z) of statistical peak of the thalamus in the PPI analysis: (10, –16, 2), Z-score: 4.63. MNI brain template: https://www.nitrc.org/projects/mricron.

Itch rating was reported at the end of six blocks of itch stimuli in the present study. No subjects reported that there was robust decay in perceived itch sensation in each block or that there was a block that they did not perceive the itch sensation. Each subject perceived similar intensity of itch sensation during the whole session. Average itch NRS rating of all subjects (mean ± SD) was 4.3 ± 1.7. As shown in Figure 1A, the itch stimuli induced significant activation of the contralateral pIns (i.e., the right pIns). In addition significant activations were observed in the PFC including the fronto-polar cortex (FPC) corresponding to the brodmann area (BA) 9/10 and ventrolateral PFC (vlPFC) corresponding to the BA 44, pre-SMA/SMA, premotor cortex (PM), secondary somatosensory cortex (SII), parietal cortex, orbitofrontal cortex (OFC), aIns, BG including the striatum and globus pallodius (Gp), anterior midcingulate cortex (aMCC), posterior MCC (pMCC), cerebellum (Table 1). No brain region showed significant correlation with itch NRS rating.

**TABLE 1:** Activationand functional connectivity during itch stimuli.

	**Activaion during itch stimuli**				**Functional connectivity during itch stimuli**			
**Brain region**	**MNI coordinate**			**z score**	**MNI coordinate**			**z score**
	**x**	**y**	**z**	****	**x**	**y**	**z**	****
FPC	32	36	22	4.33				
(BA 9/10)	32	52	22	4.32				
	20	60	20	4.41				
	44	48	2	4.3				
	−42	46	12	4.17				
	−32	36	10	4.29				
vlPFC	56	12	2	4.69	54	18	0	4.8
(BA 44)	−60	8	4	4.72	−60	2	10	5
OFC	−32	48	−18	4.57				
Premotor	48	10	50	5.59				
Pre-SMA/SMA	10	16	60	4.56	10	14	66	3.89
	6	24	44	4.24	4	16	40	4.28
aMCC	12	28	26	4.44	8	20	28	4.43
	−14	20	58	4.22	−4	24	28	4.54
pMCC	−2	−18	38	4.25				
alns	40	4	4	4.7	34	14	6	4.81
	42	16	−4	4.51	40	2	4	5.31
	42	8	−12	4.96	−40	2	8	5.49
	−36	0	4	4.8				
	−34	16	2	4.35				
	−44	10	−12	4.59				
pIns	40	−12	2	4.71				
BG	30	12	4	4.88	30	−6	6	5.34
	30	8	−8	4.65	34	14	6	4.81
	−10	10	8	4.24	34	−4	−4	5.46
					−22	2	4	4.7
					−16	4	2	4.62
Temporal	−56	2	6	4.6	40	0	−20	5.3
	54	−2	4	4.58				
	−54	−38	−16	4.59				
SII	62	−16	16	4.3	62	−22	16	4.99
	−64	−20	22	4.98	−56	−26	18	5.07
Parietal	52	−56	44	4.03				
	54	−40	26	5.19				
	−44	−54	46	3.47				
	−66	−48	20	5.03				

*FPC, fronto-polar cortex; BA, brodmann area; vlPFC, ventrolateral prefrontal cortex; PM, premotor cortex; OFC, orbitofrontal cortex; SMA, supplementary motor area; aMCC, anteiror midcingulate cortex; aIns, anterior insula; pIns, posterior insula; BG, basal ganglia; SII, secondary somatosensory cortex.*

### Functional Connectivity With the pIns During Itch Stimuli

**FIGURE 2:**
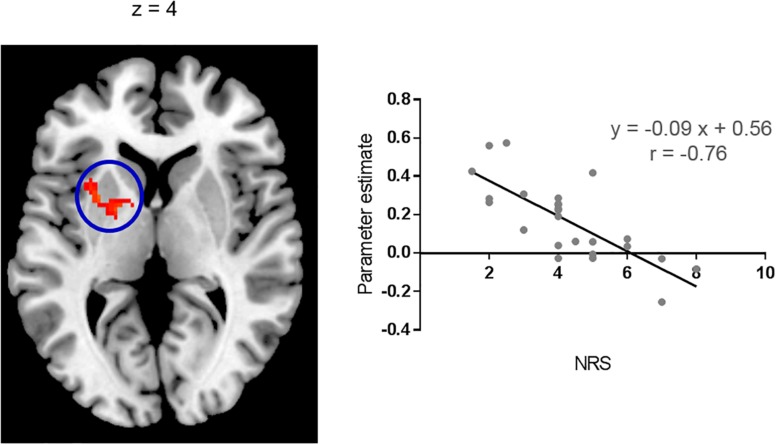
Correlation with itch NRS rating. Functional connectivity between the right pIns and left BG during itch stimuli had significant and negative correlation with itch NRS. Scatter plot: Mean parameter estimates of the cluster (cluster size: 157 voxels) and itch NRS rating. Parameter estimate reflects intensity of functional connectivity between the right pIns and left BG. MNI brain template: https://www.nitrc.org/projects/mricron.

Brain regions showing significant functional connectivity with the seed region (i.e., the right pIns) during itch stimuli were observed in the bilateral pre-SMA/SMA, aMCC, SII, aIns, BG, temporal cortex, and vlPFC (Figure 1A and Table 1). Since activation of the thalamus due to itch stimuli did not reach our cluster size threshold (i.e., FWE *p* < 0.05), this brain region was excluded in the functional connectivity analysis (see section “Materials and Methods”). However, it is important to investigate whether the thalamus and pIns increases functional connectivity during itch stimuli to confirm that the pIns used for the functional connectivity analysis receives itch-related signals from the thalamus. Thus, we conducted functional connectivity analysis focusing on the right pIns and thalamus. That is, we applied a mask image of the thalamus (PickAtlas^2^) instead of brain regions activated by itch stimuli in the second-level analysis of functional connectivity with the posterior. An applied threshold was the same as that used in other analyses (i.e., uncorrected *p* < 0.001 and FWE *p* < 0.05 for the whole brain). As shown in Figure 1B, these brain regions showed significant functional coupling during itch stimuli. Intensity of functional connectivity between the seed region (i.e., the right pIns) and left BG including the striatum [MNI coordinate of statistical peak: (−30, 6, 0), z-score: 4.3] and Gp [MNI coordinate of statistical peak: (−18, 0 6), z-score: 4.27] was significantly and negatively correlated with itch NRS rating (Figure 2). Functional connectivity between the seed region and BG within the same hemisphere (i.e., the right hemisphere) also showed negative correlation with itch NRS rating in two regions (significant for intensity threshold in both regions), however, they did not reach our cluster size threshold [The striatum: MNI statistical peak coordinate: (16, −2, 14), z-score: 3.91, *k* = 57, Correlation coefficient: *r* = −0.64; The Globus pallidus: MNI statistical peak coordinate: (26, −6, −2), z-score: 3.89, *k* = 56, Correlation coefficient: *r* = −0.68].

## Discussion

This is the first study to investigate the brain network that processes itch-related signals conveyed from the periphery to the pIns using functional connectivity MRI. Our results suggest that the functional coupling between the pIns and pre-SMA/SMA, aMCC, aIns, SII BG, is a key network to process itch. In particular, the pIns-BG pathway plays an important role in the regulation of subjective itch sensation.

### The pIns Activated by Itch Stimuli

In the present study, we observed a significant activation of the contralateral pIns: i.e., the pIns innervating the body side where itch stimuli were applied. No other location of the pIns in both hemispheres showed significant activation during itch stimuli. The location in the pIns that we observed was similar to that reported in previous studies (Drzezga et al., 2001; Leknes et al., 2007; Papoiu et al., 2012). The pIns is a major brain region receiving neural signals from the STT via the thalamus (Dum et al., 2009; Craig, 2010). In the present study, we observed significant increment of functional coupling between the pIns and thalamus during itch stimuli (Figure 2), indicating transmissions of itch-related signals from the thalamus to the pIns.

### Brain Regions Showing Significant Increment of Functional Connectivity With the pIns

In the present study, we observed significant activations due to itch stimuli in key brain regions associated with itch such as the aIns, SII, and BG. These brain regions receive fiber projections from the pIns in humans and primates (Mesulam and Mufson, 1982; Chikama et al., 1997; Cerliani et al., 2012; Ghaziri et al., 2017, 2018; Failla et al., 2017). In accordance with these anatomical projections, we observed that the right pIns showed significant increments of functional connectivity with the SII, BG, and aIns in the same hemisphere (i.e., the right hemisphere) during itch stimuli. The SII in the opposite hemisphere (i.e., the left hemisphere) also showed significant increments of functional connectivity with the right pIns during itch stimuli. The right and left hemispheres are connected to each other through the corpus callosum. A previous magnetoencephalogray (MEG) study reported that itch-related signals are transmitted from the SII in one hemisphere to that in the other hemisphere through the corpus callosum (Mochizuki et al., 2009). This transmission may explain the significant increments of functional coupling of the SII and pIns across the hemispheres during itch stimuli in the present study. In addition to the SII, functional coupling was also observed between the pIns in the right hemisphere and BG and aIns in the opposite hemisphere. Activity of the BG and insula is strongly synchronized between the hemispheres through the anterior commissure and/or corpus callosum (O’Reilly et al., 2013; Sun et al., 2015; Qi et al., 2016; Su et al., 2016). This may explain why the pIns increased functional connectivity with the BG and aIns not only in the same hemisphere but also opposite hemisphere. Another possibility may be that itch-related neural signals reached to the SII in the left hemisphere via the corpus callosum were traveled to the BG and aIns in the same hemisphere (i.e., the left hemisphere) (Chikama et al., 1997; Cauda et al., 2011; Cloutman et al., 2012). The pre-SMA/SMA and aMCC have robust anatomical connections with aIns, but not with the pIns (Mesulam and Mufson, 1982; Cerliani et al., 2012; Ghaziri et al., 2017). There is an anatomical connection between the pIns and aIns (Failla et al., 2017), speculating a possible transmission from the pIns to the pre-SMA/SMA and aMCC via the aIns.

Previous brain imaging studies reported that activity in the pre-SMA/SMA, aMCC, BG, and aIns were significantly and positively correlated with temporal change in itch sensation induced by pruritogens such as histamine and allergens (Leknes et al., 2007; Mochizuki et al., 2007). The same relationship was observed in temporal changes between activity in unmyelinated fibers provoked by histamine-induced itch and the itch sensation (Schmelz et al., 1997). These findings suggest that activity in the pre-SMA/SMA, aMCC, BG, and aIns represents or is in parallel with activity in unmyelinated fibers due to itch. These brain regions may be a key network encoding precise somatosensory stimulus features. Unlike these brain regions, activity in the SII does not show significant correlation with temporal change in subjective itch sensation (Leknes et al., 2007; Mochizuki et al., 2007). Response of the SII to itch stimuli seems to be transient or lasts only a short period (Herde et al., 2007; Mochizuki et al., 2009, 2014). An fMRI study with monkeys reported that the SII and pIns constitute distinct networks with different brain regions and these networks are joined in the process of pain via the SII-pIns connection. Perhaps, the SII may constitute another network with different brain regions in the processing of itch, and functional connectivity between the SII and pIns during itch stimuli could reflect connections between the network involving the SII and network composed of the pIns, pre-SMA/SMA, aMCC, BG, and aIns. It was reported that the SII is associated with detections of salient events or change of somatosensory stimuli (Chen et al., 2008; Yamashiro et al., 2009; Mouraux et al., 2011; Otsuru et al., 2011). The SII may play an important role in the prompt recognition of itch or changes in itch intensity so as to initiate appropriate reactions to itch, such as scratching. The FPC, PM, pMCC, parietal cortex, and cerebellum significantly activated by itch stimuli did not show significant increments of functional connectivity with the pIns during itch stimuli, indicating that these brain regions are not primarily involved in the process of itch originated from the pIns.

### Negative Correlation of Functional Connectivity With Itch NRS Rating in the BG

In the present study, physical intensity of itch stimuli were the same in all subjects (i.e., 0.35 mA). However, subjective itch sensation (i.e., itch NRS) varied from 1 to 8. This variation was negatively correlated with intensity of functional connectivity between the right pIns and left BG in the present study. That is, subjects who reported stronger itch sensation had weaker functional connectivity between the right pIns and left BG during itch stimuli. It was possible to interpret that the left BG, but not the right BG, plays an important role in regulation of subjective itch sensation. However, though it did not reach our cluster size threshold, two regions in the right BG showed that intensity of functional connectivity with the right pIns had negative correlation with itch NRS (at least significant for intensity threshold for both regions). Considering strong interhemispheric connectivity of the BG (O’Reilly et al., 2013; Qi et al., 2016), functional network composed of the pIns and bilateral BG may play an important role in regulation of subjective itch sensation.

There are some potential mechanisms that can explain the relationship between subjective itch sensation and functional pIns-BG connectivity observed in the present study. For example, the BG constitutes a large loop circuit with the thalamus and cortex related to somatosensory processing including the SI, SII, cingulate cortex, insula, prefrontal cortex, and parietal cortex (Chudler and Dong, 1995). The process of itch-related signals in the circuit may be influenced by intensity of functional connectivity between the pIns and BG, which could affect subjective itch sensation. Another putative explanation for this finding is related to a descending itch modulation. The striatum has neurons sending nerve fiber to the medullary dorsal reticular nucleus (RVM) (Borsook et al., 2010; Barceló et al., 2012). The RVM sends descending pathway to the spinal cord and modulates pain and itch (Millan, 2002; Zhao et al., 2014). Perhaps, increased functional connectivity between the pIns and BG could activate the descending neural pathway and suppressed itch at the spinal level.

Another interesting mechanism associated with functional pIns-BG connectivity may relate to the desire to scratch. The BG is a core of motivation and cravings (Vijayaraghavan et al., 2008; Adam et al., 2013; Berridge and Robinson, 2016; Volkow et al., 2017) and also associated with the desire to scratch provoked by itch (Leknes et al., 2007; Mochizuki et al., 2014). The network composed of the BG, aIns, and anterior cingulate cortex (ACC) plays an important role in regulation of craving (Naqvi et al., 2014). It was reported that this network is involved in the desire to scratch provoked by viewing pictures depicting itch (Mochizuki et al., 2013). Considering that the desire to scratch is a major component of the itch sensation, functional connectivity between the pIns and BG may influence subjective itch sensation by modulating connection of the BG with the aIns and ACC.

### Limitations

In the present study, we investigated a functional network to process itch using mechanical itch stimuli. However, it was uncertain whether chemical itch such as histamine- and cowhage-induced itch constitute the same network as we observed in the present study. Brain regions activated by electrical itch stimuli and functional network originated from the pIns observed in the present study were similar to many sites in pain studies (Apkarian et al., 2005; Peltz et al., 2011). As we did not examine pain perception in the present study, future studies should compare brain activations and functional connectivity between itch and pain to identity itch selective brain network, which would further advance our understanding of the brain processing of itch. This study was conducted in healthy subjects. It would be of interest to examine whether functional connectivity of the pIns differs between healthy subjects and chronic itch patients.

## Conclusion

In the present study, we observed that the pIns increased functional connectivity with key brain regions associated with itch such as the pre-SMA/SMA, aMCC, aIns, SII, and BG during itch stimuli. In particular, it was suggested that the functional connectivity between the pIns and BG is important in regulation of subjective itch sensation in healthy. These findings support the central role of functional network originated from the pIns in itch perception.

## Ethics Statement

This study was carried out in accordance with the recommendations of Ethics Committee of the National Institute for Physiological Sciences with written informed consent from all subjects. All subjects gave written informed consent in accordance with the Declaration of Helsinki. The protocol was approved by the Ethics Committee of the National Institute for Physiological Sciences.

## Author Contributions

HM conceptualized the overall study, collected the data, analyzed the data, participated the analyses of the MRI data, and wrote the manuscript. LH analyzed the fMRI data and wrote the manuscript. GY discussed the results and wrote the manuscript. NS supervised the fMRI data analysis, discussed the results, and wrote the manuscript. RK discussed the results and wrote the manuscript. All authors read and approved the manuscript.

## Conflict of Interest Statement

The authors declare that the research was conducted in the absence of any commercial or financial relationships that could be construed as a potential conflict of interest.

## Footnotes

1 http://www.fil.ion.ucl.ac.uk/spm

2 http://fmri.wfubmc.edu/software/pickatlas
